# Regional and temporal coordinated mutation patterns in SARS-CoV-2 spike protein revealed by a clustering and network analysis

**DOI:** 10.1038/s41598-022-04950-4

**Published:** 2022-01-21

**Authors:** Surendra S. Negi, Catherine H. Schein, Werner Braun

**Affiliations:** 1 Sealy Center for Structural Biology and Biophysics, Department of Biochemistry and Molecular Biology, The University of Texas, Medical Branch, 301 University Blvd, Galveston, TX 77555-0304 USA; 2 Institute for Human Infections and Immunity (IHII), The University of Texas Medical Branch, Galveston, TX 77550 USA

**Keywords:** Computational biology and bioinformatics, Evolution, Immunology

## Abstract

SARS-CoV-2 has steadily mutated during its spread to > 300 million people throughout the world. The WHO has designated strains with certain mutations, “variants of concern” (VOC), as they may have higher infectivity and/or resist neutralization by antibodies in sera of vaccinated individuals and convalescent patients. Methods to detect regionally emerging VOC are needed to guide treatment and vaccine design. Cluster and network analysis was applied to over 1.2 million sequences of the SARS-CoV-2 spike protein from 36 countries in the GISAID database. While some mutations rapidly spread throughout the world, regionally specific groups of variants were identified. Strains circulating in each country contained different sets of high frequency mutations, many of which were known VOCs. Mutations within clusters increased in frequency simultaneously. Low frequency, but highly correlated mutations detected by the method could signal emerging VOCs, especially if they occur at higher frequency in other regions. An automated version of our method to find high frequency mutations in a set of SARS-COV-2 spike sequences is available online at http://curie.utmb.edu/SAR.html.

## Introduction

Coronavirus infection (COVID-19), caused by severe acute respiratory syndrome coronavirus 2 (SARS-CoV-2)^1,2^ has now spread throughout the world, causing over 300 million confirmed cases, and 5 million deaths worldwide (https://coronavirus.jhu.edu/). The spike protein of SARS-CoV-2 is 80% identical to the SARS virus which caused many deaths in Asia around 2003^3^ and has a similar 3D-structure^4^. Both SARS distinguish themselves from other β-coronaviruses by using the human angiotensin-converting enzyme 2 (ACE2) receptor to bind cells^5^. The SARS-CoV-2 spike protein is recognized by antibodies isolated from SARS-CoV-1 survivors^6–8^ but it may not be neutralized by the same antibodies. Although the current vaccines are all based on one isolate (Wuhan, 2019), SARS-CoV-2 has subsequently evolved into a mixture of strains during millions of human infections. Almost all currently circulating strains contain one change from the original Wuhan sequence, D614G, which may alter the viruses ability to evade the immune response^9,10^. Strains containing other mutations that occur regionally at high frequency have been recognized as “variants of concern” by the World Health Organization (https://www.cdc.gov/coronavirus/2019-ncov/variants/variant-info.html#Concern), as they are not neutralized as efficiently by antibodies in sera of convalescent patients or vaccinees. These include strains B.1.1.25 in Australia, B1.1.7 in UK^11^, P.1 and P2 in Brazil^12^, B1.351 in South Africa^13^, B1.617 in India^14,15^ and B.1.526 in the US^16,17^. The VOCs originally identified in the UK, South Africa, and Brazil are also now common in the US^18,19^. Treatment plans must take into account strain information, as for example the South African variant B.1.351 is resistant to neutralization by convalescent plasma from COVID-19 survivors and monoclonal antibody treatments^20–22^. In vitro studies with pseudo-viruses indicate that sera of vaccinated individuals have reduced ability to neutralize other common variants, including E484K and N501Y^23,24^, suggesting that these should be included in future vaccines.

While VOCs are characterized by a pattern of mutations, there is no clear theory about which changes are compensatory or additive in their ability to change the infectivity, pathogenesis or resistance of the virus. For example, our preliminary analysis of 1.8 million SARS-CoV-2 spike sequences from the GISAID database revealed over 350 amino acid variants, about 100 of which occur in the receptor binding domain (RBD)^25,26^. To systematically identify groups of mutations that occur together, we developed a new bioinformatics approach to characterize spatial and temporally correlated mutations within the spike protein based on their similar frequency of occurrence. The highest frequency clusters detected by our automated screening method included all previously known VOCs. In addition, regional and temporal analysis revealed other clusters that were increasing in frequency and could be emerging VOCs. The data also suggest how future vaccine design could be tailored to the local strain ensemble.

## Results

### Major mutations in the spike protein and relations to VOCs

We collected two groups of full-length spike protein sequences from the GISAID database; a group 1 with sequences from all countries, and a separate group (group 2) from 36 countries where COVID-19 was spreading rapidly. The top ten mutations found in the group 1 and for twelve individual countries are listed in Table 1. Additional data for the top 16 mutations in twelve individual countries are shown in supplementary material (Table Sup.T1), and a complete list of mutations for all 36 countries is shown in an MS Excel file in the supplementary material (Table Sup T2). The top eight of these mutations in group 1, i.e. D614G, P681H, N501Y, T716I, D1118H, A570D, S982A, and A222V are also found as high frequency mutations in most individual countries. Especially, the D614G mutation, found to be the dominating mutation in the first viral variant B.1.1.7^10,27^ is also the top ranked mutation in all individual countries. The D614G mutation is responsible for increased viral transmission^10,28^. The second major mutation found in most countries, P681H, which increases the viral fitness in the fusion process, is located near the furin cleavage site between S1 and S2 in the spike protein^29^. In some countries e.g. India, this residue was mutated to P681R. Other major mutations such as N501Y, result in increased infectivity as the mutated residue increases the affinity for the receptor binding domain (RBD) to its receptor ACE2^30^, or the mutation E484K that reduces the immune response^20,31–33^. Other highly ranked mutations in each country are more country specific and may evolve with time and geographic locations under selective pressure. For example, the mutations, S13I and W152C found in the US, could interfere with neutralizing antibodies binding to the N-terminal domain (NTD), as antibody interacting residues W152 and R246 have been identified for the neutralizing antibody 4A8 for SARS-CoV-2^34^.

**Table 1:** The top 10 mutations (after the D614G mutation, the first divergence from the “Wuhan 2019” sequence which arose rapidly and is present throughout the world at high frequency) in the spike protein listed in order of their frequency in sequences from all countries, and twelve different countries.

Country	Mutation rank									
	1	2	3	4	5	6	7	8	9	10
All*	D614G	P681H	N501Y	T716I	D1118H	A570D	S982A	A222V	L18F	S477N
USA	D614G	P681H	N501Y	T716I	A570D	D1118H	S982A	L452R	W152C	S13I
UK	D614G	P681H	N501Y	T716I	A570D	S982A	D1118H	A222V	L18F	L5F
France	D614G	N501Y	T716I	P681H	A570D	S982A	D1118H	S477N	A222V	E484K
Germany	D614G	N501Y	P681H	S982A	T716I	D1118H	A570D	A222V	L18F	S98F
Spain	D614G	P681H	T716I	N501Y	A570D	S982A	D1118H	A222V	D138Y	L18F
Italy	D614G	N501Y	P681H	T716I	D1118H	S982A	A570D	A222V	P272L	A262S
China	D614G	S12F	H49V	M153T	S50L	A688N	D1084E	Q498H	V1228I	F32S
India	D614G	P681R	E484Q	L452R	N440K	G142D	Q1071H	E154K	N501Y	Q677H
South Korea	D614G	N501Y	P681H	T716I	D1118H	A570D	S982A	L452R	S13I	W152C
Japan	D614G	M153T	Q675H	E484K	W152L	G769V	P681H	L54F	Q677H	G184S
Brazil	D614G	V1176F	E484K	N501Y	L18F	H655Y	P26S	D138Y	T20N	T1027I
South Africa	D614G	A701V	E484K	D80A	K417N	N501Y	D215G	L18F	R246I	A688V

The most common alterations are P681H (near the furin cleavage site that is a major distinction between SARS-CoV-2 and SARS) and N501Y (in the ACE2 binding region of the RBD).

*All SARS-CoV-2 sequences in the GISAID database.

Our list of top mutations correlates with the signature sequences of the major VOC as defined by an expert group of the WHO. For example, the major mutations we found for the UK, D614G, P681H, N501Y, T716I, A570D, S982A and D111H are the signature of the Alpha variant (B.1.1.7). The top mutations (D614G, A701V, E484K, D80A, K417N, N501Y and D215G) in South Africa are the signature of the Beta variant (B.1.351). Of course, in most of the countries a mixture of different variants is circulating, as seen for the US, where the top mutation clusters are from the Alpha variant, and the next most frequent mutations, S13I, W152C, and L452R from the Epsilon variant (B.1.429).

### Deletion pattern of the spike protein sequences

The deletions in the N-terminal domain of the spike protein at H69, V70 and Y144^11,23,35^ were first found in sequences from UK and may allow the virus to evade the immune response^36^. However, from December on-wards we found this deletion also more frequently in sequences obtained from US, Italy, Germany, Spain, France and other European countries but less in sequences obtained from Brazil, India and South Africa.

### Location of the high frequency mutations on the 3D structure of the spike protein

**Figure 1:**
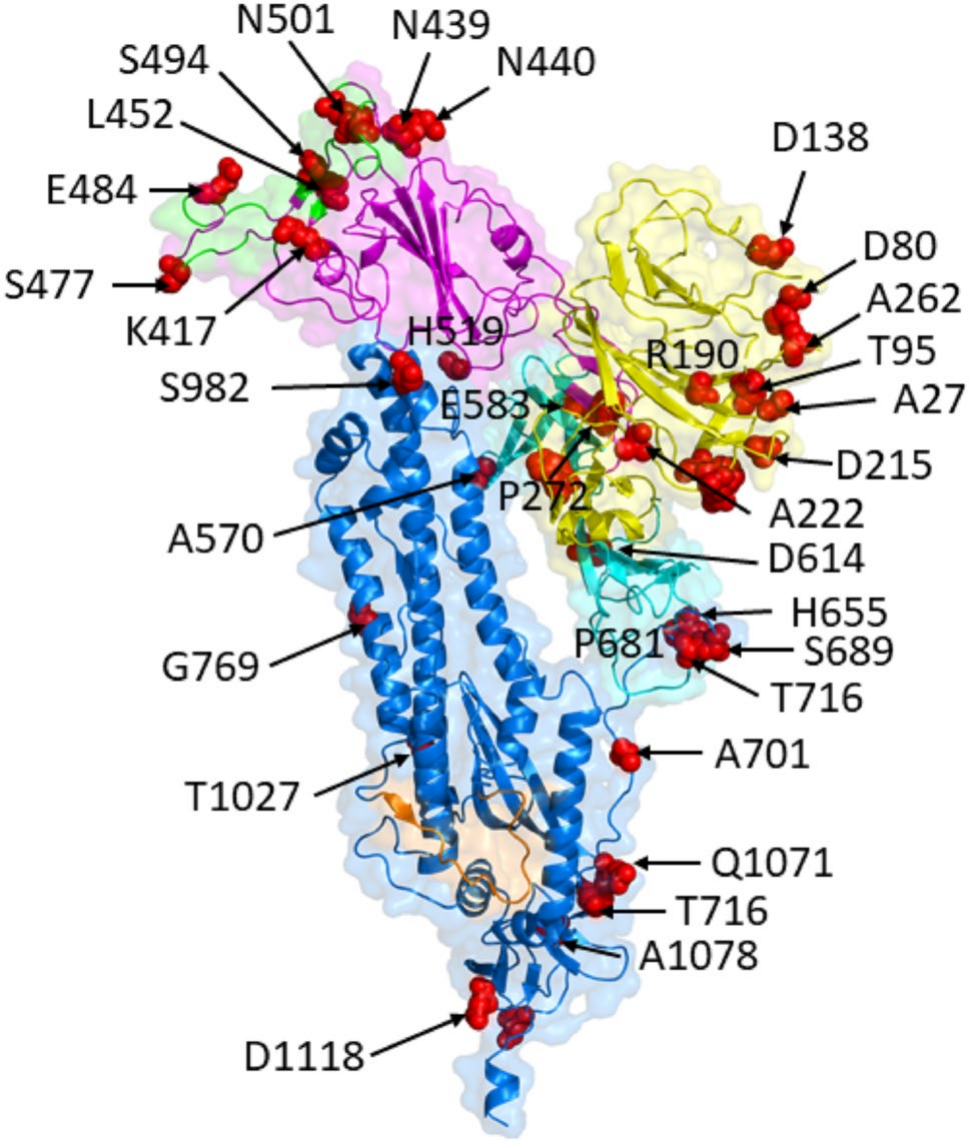
Most frequent mutations (red color) in the SARS-CoV2 spike protein determined by clustering and alignment methods. The two subunits of the S protein are S1, which contains the RBD (magenta) and the N-terminal domain (yellow), and S2 (marine). The ACE2 binding site on the RBD is shown in green, and the fusion peptide in orange. Most high frequency mutations were detected in the RBD and the NTD.

The Spike protein is synthesized as a single chain protein which is then cleaved by a furin protease into two subunits, known as S1 and S2 (Fig. 1). The N-terminal domain (NTD) and the receptor binding domain (RBD) of the S1 protein are important areas for immune recognition and receptor binding^37^. The RBD is both a target for vaccine^38^ and diagnostic^39^ design. The helical S2 subunit undergoes large conformational changes during the fusion process. The 3D structure of the peptidase domain of ACE2 in a complex with the spike protein (PDB file: 6M17)^40^ shows that ACE2 interacts with at least 22 amino acids in the RBD, including several frequently mutated residues detected by our method: K417, S494, N501, L454, S477 and E484. Compounds that inhibit the interaction between the RBD and ACE2 may be useful treatments for SARS-CoV-2 infection^41^.

Our sequence analysis showed that RBD positions K417, S494 and N501, which form contacts with ACE2, and other three mutations at L452, S477 and E484 are located close to the ACE2 interface, and may play an important role in binding to receptor and antibodies^8,42,43^. In addition, this site is targeted by several antibodies as reported recently^44–46^. Most of the mutations on the spike protein found by our analysis are in the RBD and N-terminal domain of the S1 protein, or close to the fusion peptide (e.g. T716, T1027, and Q1071) and CD domain (D1118) of S2 protein. Most of the mutated residues in the RDB, except N501, are surface exposed in the PDB id 6M17 and 6BSB structures according to GetArea^47,48^.

Notably, there were distinct mutation signatures for individual countries. For example, in India we found a major mutation at the P681 position but with an R (9.71%) instead of the H mutation. The R mutation is also found in the Delta variant (B.167.2). This variant is of considerable public concern, as it is increasing with a high rate in many countries, including the US, due to its increased transmissibility and immune evasion^49,50^.

### Correlation analysis of frequency mutation indices across the 36 countries

**Figure 2:**
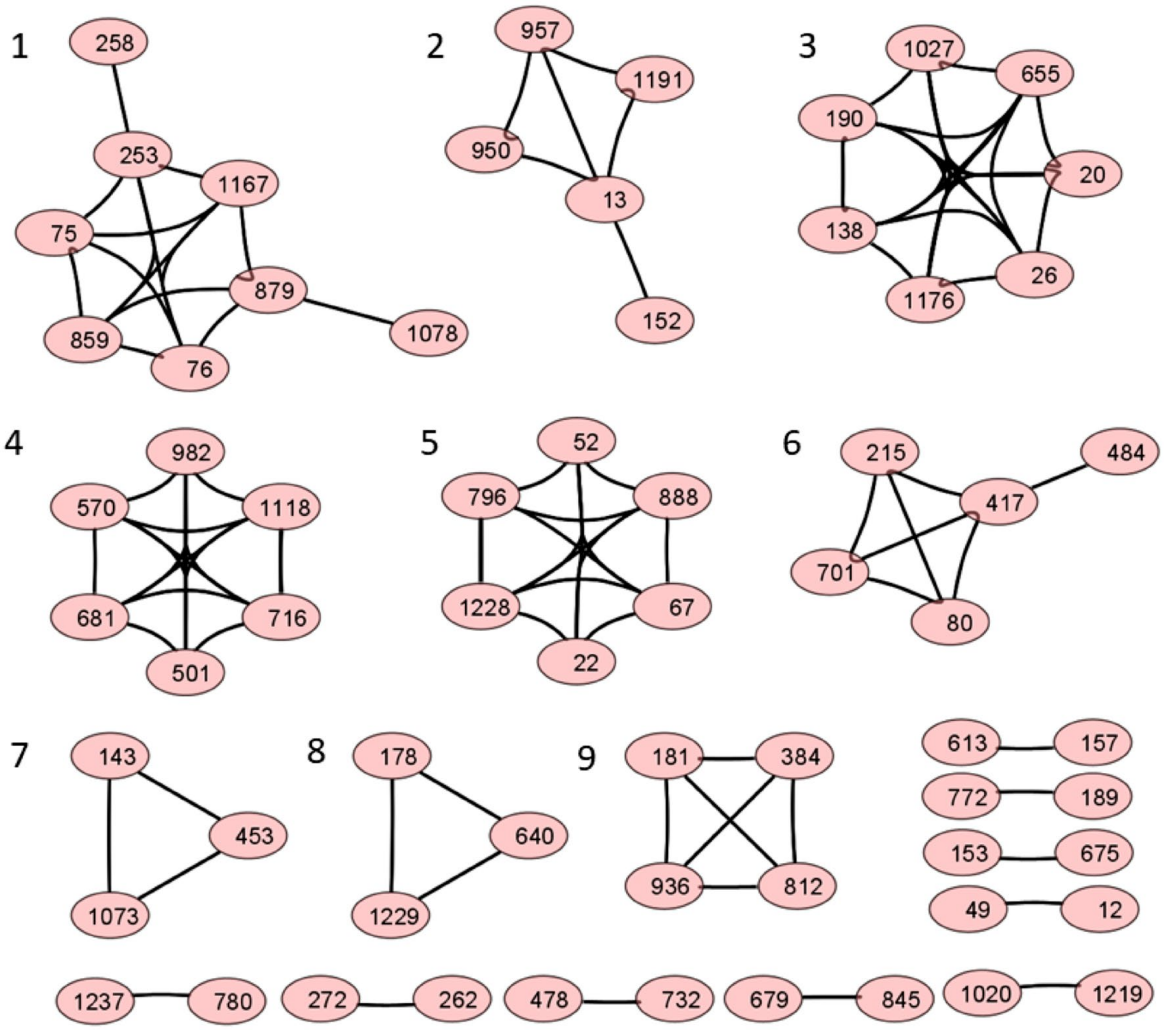
A network analysis of mutation data in sequences from 36 countries yields nine major clusters of amino acids, suggesting mutations at certain positions evolved together. Each node in the network represents an amino acid position on the SARS-CoV2 wild type sequence. Several clusters have mutations that are characteristic of variants of concern, e.g. amino acids (N501, P681, T716, S982, A570 and D1118) in cluster 4 represent signature mutations in the Alpha variant, while those in cluster 6 correspond to the Beta variant.

**Figure 3:**
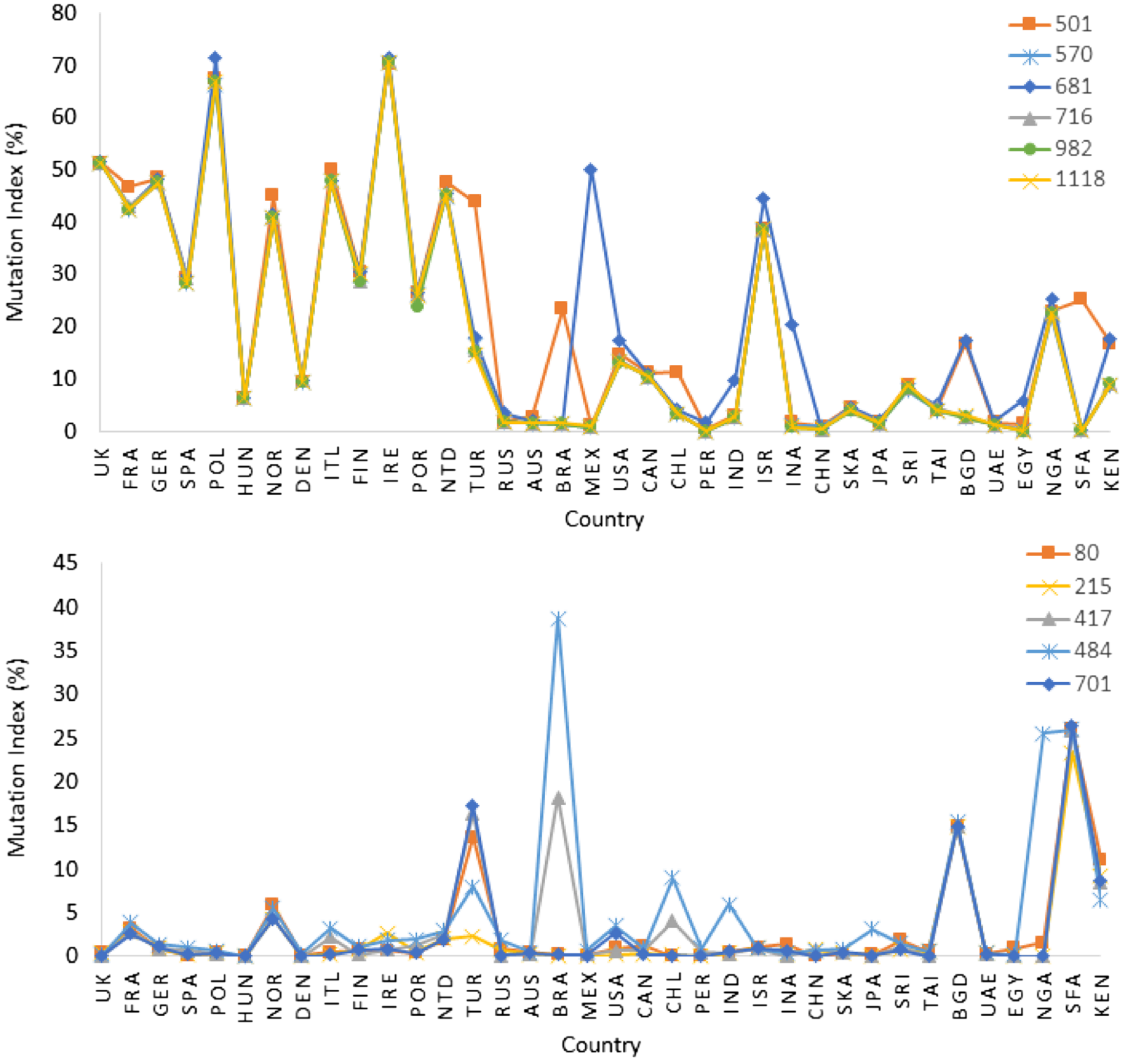
Profile of discrete mutations frequency across different countries as found in (**a**) amino acids in cluster 4 and (**b**) cluster 6, of Fig. 2.

We performed a correlation analysis, visualized with Cytoscape, of the top 100 mutations in the spike protein by calculating the percentage mutation frequencies in 36 different countries^51^. Using a correlation coefficient cutoff of 0.7, we obtained nine major clusters that contain three or more amino acids (Fig. 2). We empirically used a relatively low threshold of 0.7 to find mutations that arise as groups in different countries, as the number of sequences from each country varies. The clusters found by this analysis are characteristic for clusters that emerge in different geographic locations. For examples the mutations found in cluster 4 (N501Y, A570D, P681H, T716I, S982A, D1118H) occur mainly in European countries, whereas mutations in cluster 6 (D80A, D215G, K417N, E484K, A701V) are mostly from South American countries and South Africa, as seen in Fig. 3.

The mutations observed in cluster 4 coincide with the signature mutations in the Alpha variant as defined by the WHO. Cluster 4 also contains six major mutations with a frequency index above 10% in most countries. These mutations first appeared during the first COVID-19 wave in 2020 and were also present in sequences from other countries but with low frequency (less than 10%). Similarly, the mutations in cluster 6 are characteristic for the Beta variant, mutations in cluster 3 for the Gamma variant, mutations in cluster 2 correspond to the Epsilon variant, and mutations in cluster 5 coincide with the signature mutations of Eta. The mutation profile of the amino acids in the spike protein for cluster 4 and 6 over all 36 countries are shown in Fig. 3. The six mutations of the Alpha variant appeared together with highly similar frequencies in each country, leading to a high correlation coefficient, although the frequencies vary across the 36 countries. The plot also shows the dominance of the Alpha variant in European countries and of the Beta variant in South Africa.

Our cluster analysis thus automatically detected five major variants of SAR-CoV-2 and their signature mutations. In addition, we also found a few small clusters that are either country specific or may be due to undetected COVID-19 positive persons traveling to these countries. For example, the mutation S13I in cluster 2 was only present in sequences obtained from US, Mexico, Sri Lanka and Taiwan, while mutations D253G and T859N in cluster 1 were present in sequences from US, Chile and Peru.

### Evolution of the mutations in the spike protein over time

**Figure 4:**
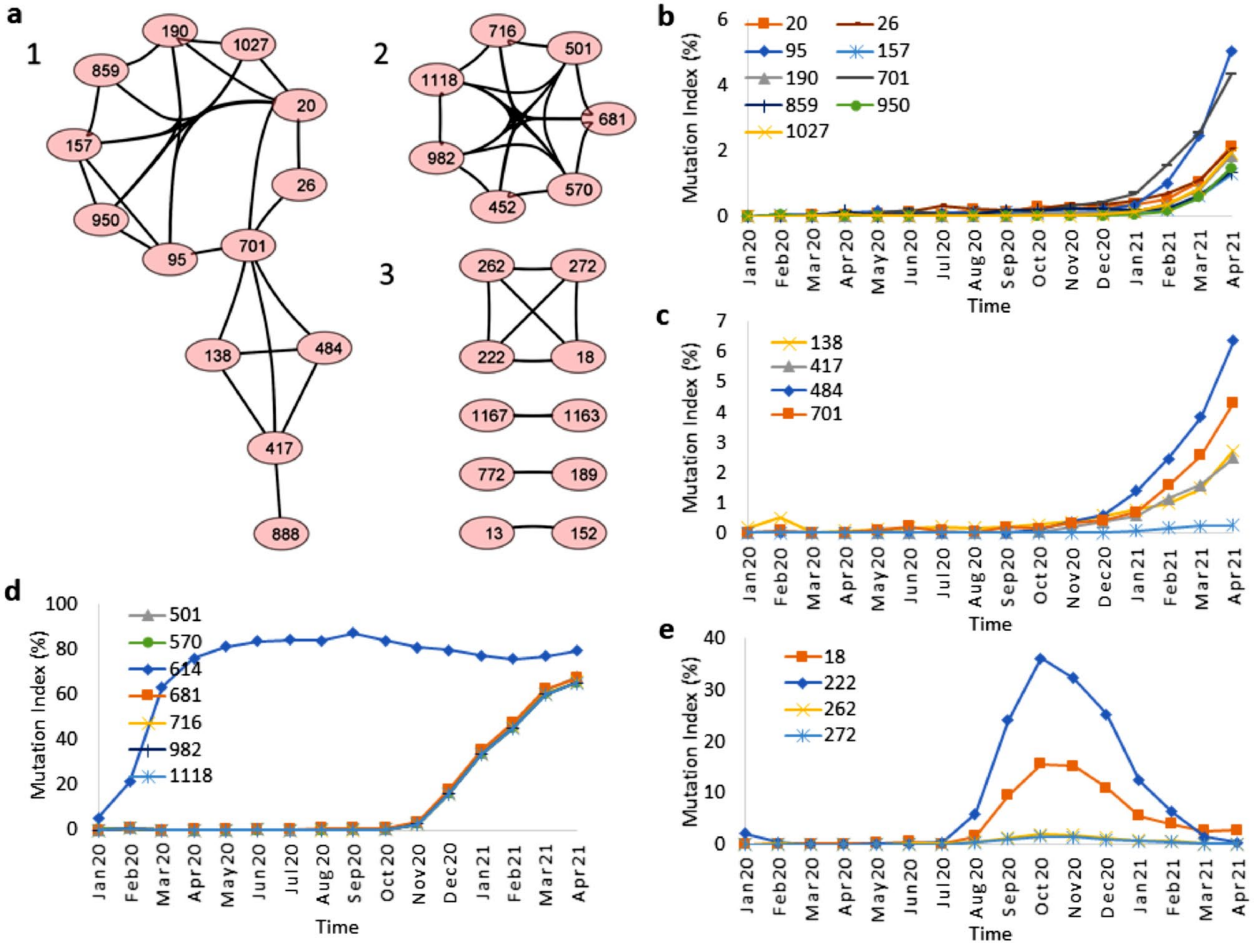
Evolution over 18 months of mutation signatures across all countries. (**a**) Three major clusters, marked as 1, 2 and 3, of mutations that each have a similar evolution profile. (**b**) and (**c**) Time evolution of mutations in cluster 1. These mutations have a low but increasing mutation index. (**d**) Mutations at residue positions in cluster 2 including the singular D614G mutation. These mutations are still present in April 2021with high frequencies. (**e**) Mutations from cluster 3. These mutations, with increasing presence from July 2020 to Nov 2020 and gradual decay after that, may have played a significant role during the second wave of SARS-CoV-2.

In order to understand the evolution of the spike proteins from the first Wuhan sequence, we calculated frequency indices of the mutations for each month, using all sequences in group 2 combined. Correlations of the frequency indices of the mutations over time and cluster analysis (cutoff value 0.98) with Cytoscape revealed three unique clusters of mutations with a high correlation coefficient (Fig. 4a). The mutations from cluster 1 increase in frequency after Jan 2021 (Fig. 4b, c), and are still circulating in the Beta and Gamma coronavirus variants. The frequencies of mutations in cluster 2 increased suddenly after Nov 2020 and became fixed in the virus profile through April 2021 (Fig. 4d). The mutation D614G is an outlier and displays an earlier unique adaption in the virus population. In contrast, frequencies of mutations in cluster 3 increase after July 2020 and then decrease after Nov 2020 (Fig. 4e).

### Highly correlated mutation patterns also appear in individual countries

To reveal the potential role of specific mutation patterns in the different stages of COVID-19 outbreaks in the individual countries, we repeated the correlation and cluster analysis for the individual countries. We show here the results for the UK, US and India. Mutation data related to South Africa, Brazil, Italy and Spain are discussed in the supplementary material.

**Figure 5:**
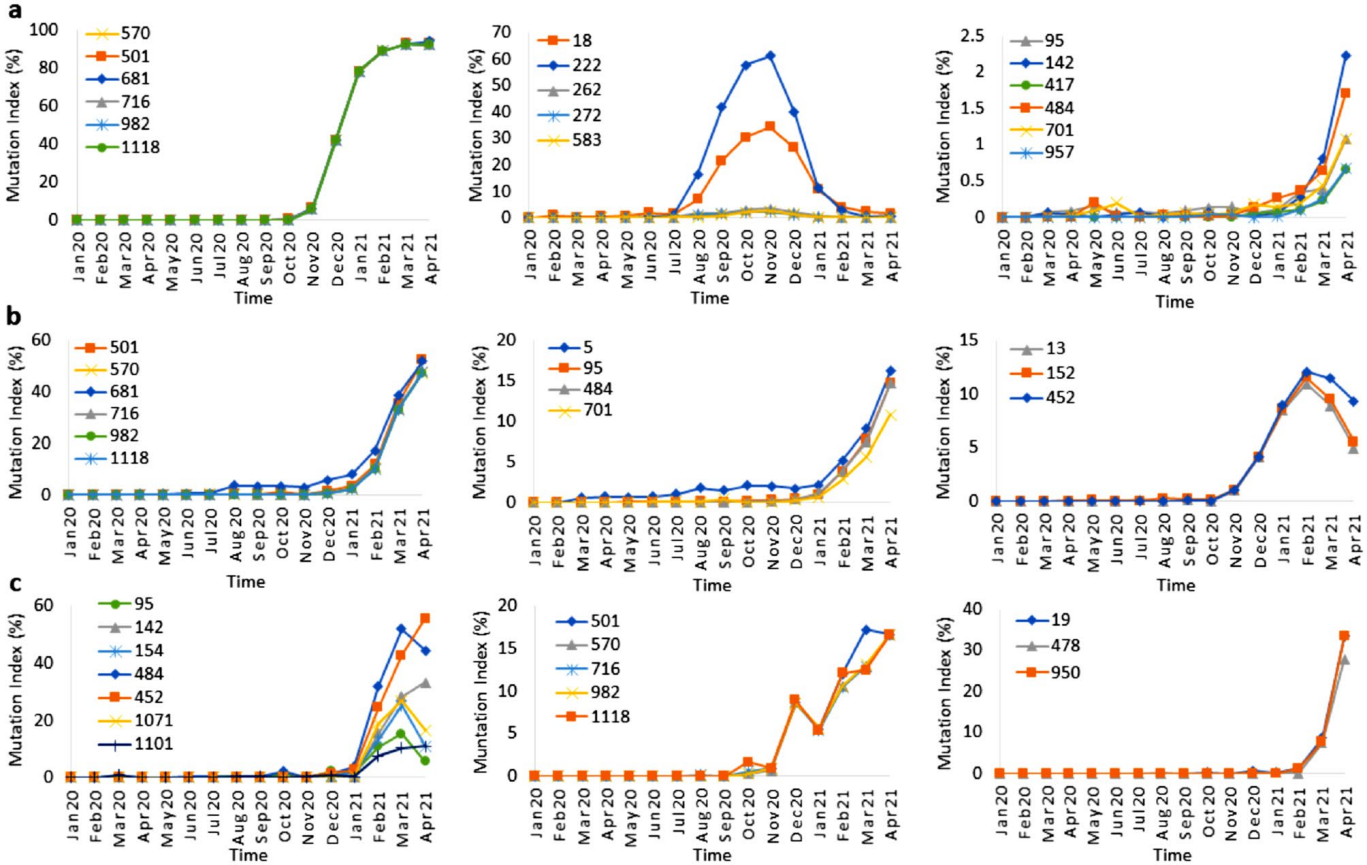
Mutation index profile from (**a**) UK, (**b**) US and (**c**) from India for SARS-CoV-2 between Jan 2020 to Apr 2021. The data for the D614G is not shown here as it is a dominant mutation since the onset of the COVID-19 pandemic, as shown in Table 1.

*United Kingdom* For the UK, a cutoff value of 0.9 identified three clusters, each having a different mutation profile with time (Fig. 5a). The first cluster of the mutations N501Y, A570D, P681H, T716I, S982A, and D1118H, the signature of the Alpha variant B.1.1.7, sharply increased in frequency in Oct–Nov 2020 through April 2021, the last month of our analysis. A second cluster, L18F, A222V, A262S, P272L and E583D peaked in frequency in Nov. 2020 and gradually dropped to its Jun 2020 level in Feb 2021. A third cluster (T95I, G142D, E484K, A701V, Q957R and K417N), has some mutations in common with the Kappa variant (B.1.167.1) first found in India (E484Q mutation), represented only 1–2% of the UK sequences in April, but is gradually increasing in the UK. Thus, our combination of correlation and network analysis can detect mutation clusters with low frequencies and an emergence potential.

*United States* The cutoff value was increased to 0.98 to obtain well separated clusters of the US data. A high frequency cluster with N501Y, A570D, P681H, T716I, S982A, D1118H and a moderate (10–20%) one, with L5F, T95I, E484K and A701V mutations dominated the data (Fig. 5b). Recent New York and California variants B.1.526 and B.1.427/B.1.429 all contain E484K, with the CA variant also characterized by S13I, W152C and L452R (third cluster). The CA mutations first appeared in Oct, peaked in Jan–Feb 2021, and decreased in Mar–April21.

*India* Using a 0.98 cutoff value we found one large cluster with amino acids having a low mutation frequency index (< 2%, hence not considered as dominant mutations) and three small clusters with high frequency mutations (Fig. 5c). The first cluster contains mutations that suddenly increase after Jan. 21 (Fig. 5c, left panel). Some of these mutations, such as G142D, E154K, L452R, E484Q and Q1071H are major mutations and may be responsible for the second wave of COVID-19 in India. Others, such as T95I, E154K, E484Q, and Q1071H, decrease in Mar–Apr 2021 while G142D and L452R are still increasing after Feb. 21. The second cluster contains the major mutations of the clusters found in the UK and US. The mutations of the third cluster T19R, T478K, and D950N (Fig. 5c right panel) dramatically increase after Feb 2021 (Fig. 5c). These mutations together with G142D, L452 and P681R are the hallmark of the Delta variant (B1.617.2), thus indicating the importance of time correlation of mutations for indicating potential emerging variants of SARS-CoV-2.

## Discussion

Since the emergence of the SARS coronavirus in Wuhan (China) in December 2019, an explosion of sequence data for SARS-CoV-2 has been collected by many research groups. The GISAID^52^ database, first established for sequence data for influenza outbreaks, expanded to become the major database for accessing genomic data of circulating SARS-CoV-2 sequences. We show here that our method can use this data to follow the evolution of the virus globally or within individual countries.

Our automated method identified clusters of mutations as a function of time and locations directly from the sequence data base. We also showed that our method is potentially useful to predict future emerging variants of SARS-CoV-2, which can be directly characterized by specific mutation patterns, independent from the use of one of the different nomenclature systems which can be confusing. The US Center for Disease Control and Prevention (CDC) distinguishes the different variants by Greek letters while the Nextstrain team (https://nextstrain.org/sars-cov-2) suggests using 18 major clades as from 19 A to 21F 19B, and 20A-20I. The PANGOLIN system^53^ uses a dynamic lineage system with two clades A and B that are further divided in a hierarchical system with further subgroups with a numerical nomenclature, such as B.1.1. For example, the VOC first detected in the UK is referred to by the CDC as the Alpha variant and in the PANGOLIN system as lineage B.1.1.7^11^. Other VOCs from South Africa are denoted as B.1.351^22^, from Brazil as P.1^54^, from California as B.1.429^18^, and from India as B.1.617^14^.

The automated bioinformatics tool we introduce here can help in identifying the major mutation patterns, not just according to their individual occurrence but also to their correlations with other mutations. Our approach, combining correlation and network analysis revealed patterns of distant mutations that occur together, and identified several known VOCs of high importance identified by the CDC. Particularly of interests are the VOCs that might threaten the future use of vaccines based on the original SARS-CoV-2 genome^35,55,56^. We show results of the method here for the spike protein, the major antigen of SARS coronaviruses, but the method can also be useful for other proteins of SARS-CoV-2 and for correlations across different genes.

## Methods

### Sequence analysis

We downloaded all SARS-CoV-2 spike sequences from the GISAID database (April 2021 release)^52^ that contains 1,247,171 SARS-CoV-2 spike sequences archived during the last 16 months until April 2021. All sequences < 1000 residues in length or those with > 100 unknown or missing residues (marked as X) were removed from the data set, to yield 1,180,646 full-length spike protein sequences over all countries (group 1). A separate group (group 2) was selected with spike protein sequences from 36 countries where COVID-19 was spreading rapidly. The spike protein sequences in group 1 and group 2 were analyzed in each month to determine the mutation frequency for each residue. The CD-HIT program^57^ (version 4.8.1) was used to cluster the sequences from all groups using a 100% sequence identity. The 100% sequence identity cutoff was used to find all sequences that have at least one mutation. After clustering, one representative sequence from each of the top 1000 clusters (if available, otherwise the maximal number of observed clusters was used) was selected. In each data set, the representative set of sequences were aligned with the spike protein sequence isolated from Wuhan (GenBank id QHR63260.2). A multiple sequence alignment was generated using the MUSCLE program^58^ and the data were analyzed to obtain the mutation frequency (*P*_i_) at each position in the sequence alignment. The *P*_i_ is defined as

1 $$\begin{array}{c}{P}_{i}=100\frac{\sum {N}_{i}}{Total}\end{array}$$

where *N*_*i*_ is the total number of sequences having that mutation in the cluster, and the *Total* is the number of all sequences in the clusters used in the alignment.

### Correlation analysis

In order to characterize the correlations between different amino acid mutations, we calculated the correlation coefficients (r_xy_) between the mutations as

2 $${\mathrm{r}}_{\mathrm{xy}} =\frac{n\sum {x}_{i}{y}_{i}-\sum {x}_{i}\sum {y}_{i}}{\sqrt{n\sum {x}_{i}^{2}-{(\sum {x}_{i})}^{2} }\sqrt{n\sum {y}_{i}^{2}-{(\sum {y}_{i})}^{2}}}$$

where x_*i*_ and y_*i*_ are the mutation frequency (Pi) of the two mutations. To determine the geographical correlations, the analysis was performed for the indices of the mutations across the 36 countries (i = 1,…,36).

The time correlations (i = 1,…,16) were calculated across the 16 months from Jan 2020 to April 2021 for the sequence data for all countries, and separately for each individual country.

### Network analysis

We used Cytoscape^51^ to cluster and visualize the data obtained from the correlation analysis. For this purpose, we calculated the correlation between all rows where each column in a row represents the mutation frequency index in a country or in a month. For visualization of the correlation, the data was formatted into a three-column format where the first two columns represent the two mutations, and the third column represents their correlation coefficients. The data was clustered using Cytoscape and the clusters were visualized using a circular layout.

## Conclusion

In the current work we analyzed over a million sequences of the SAR-CoV2 spike protein to understand their evolution and relationship between various variants circulating worldwide. Using clustering and network analysis, we showed that some of these mutations evolved together and have been shown to have an important impact on transmissibility, receptor binding or immune escape. Mutations with high frequency indices may reflect virus adaptation and can impact a virus phenotype. Using our new computational analysis, we reported several instances where the results of the correlated mutation patterns over several countries or over time coincide with signature mutations of emerging VOCs, in countries such as UK, US, India and Brazil. We also detected mutations that may be future VOCs. Our tool could be also useful for other researchers wishing to detect specific mutation patterns in other areas of the SARS-CoV-2 proteome.

## Supplementary Information

Supplementary Information.

## Data Availability

Data used for the analysis are available from the GISAID database (https://www.gisaid.org/). The results from the complete frequency analysis are available as supplementary material. Additional material is available online at the, http://curie.utmb.edu/COVID19/ and the webserver to find VOC’s at http://curie.utmb.edu/SAR.html.
