# Supplementary material for: Regional and temporal coordinated mutation patterns in SARS-CoV-2 spike protein revealed by a clustering and network analysis
Source: Sci Rep. 2022 Jan 21;12:1128. doi: 10.1038/s41598-022-04950-4 (PMC8782831; doi:10.1038/s41598-022-04950-4)
Supplement: Supplementary file 1 — Supplementary Information. [file 41598_2022_4950_MOESM1_ESM.docx]

**Sections for Supplemental Material 1.** Results obtained from South Africa, Brazil, Italy and Spain are discussed in the following section.

**Brazil:** The sequences contain 3 major mutations, D614G, V1176F and E484K, with N501Y, L18F, H655Y, P26S, D138Y, T20N, Y1027I, R190S and K417N. Using a cutoff value of 0.95, we found one big cluster and several small clusters. Cluster 1 contains most of the major mutations and shows a spike in Nov2020. Cluster 2 shows a similar pattern but with reduced frequency index while cluster3 (S689I and M153T) shows a small peak in Dec20-Jan21 which gradually decays after that (Fig. S1a).

**South Africa:** Based on monthly data, the first mutation peak of COVID19 appeared in sequences deposited after Sep2020 with little change after Jan2021. Small clusters grouping S939F, S255F, M153I, and F175S appeared in Oct2020, while two additional mutations A653V, A688V, appeared in Jun-July 2020 (Fig S.1b) and gradually disappeared.

**Italy:** Using a cutoff of 0.98, we obtained three clusters. Amino acids in cluster1 (N501Y, P681H, T716I, D1118H, A570D, and S982A) show a rapid increase in mutations after Nov2020 onwards. Amino acids in cluster 2 (P26S, K417T, H655Y, T20N, T1027I and R190S) show similar mutation pattern but with less frequency (not shown). After reducing correlation threshold value to 0.96, we obtained a new cluster. Amino acids in this cluster shows a rapid mutation in A222V, P272L and A262S from Aug 2020, which peaked around Nov-Dec2020 and then decreases in Feb-Mar2021. Third cluster was obtained at 0.80 cut-off value where mutation S477N, Q675H and V1104L increases from Sep-Nov2020 then gradually decrease after Jan2021 (Fig S2a).

**Spain:** Monthly mutation data pattern of from Spain was similar to US. The first cluster shows a big mutation spike in N501Y, P681H, T716I, A570D, S982A, and D1118H from Nov20 onward while other groups are dominated by large number of mutations that increases from Jan 2021(Fig S2b).

Fig S1. Mutation Index data obtained from a) South Africa, and b) Brazil.


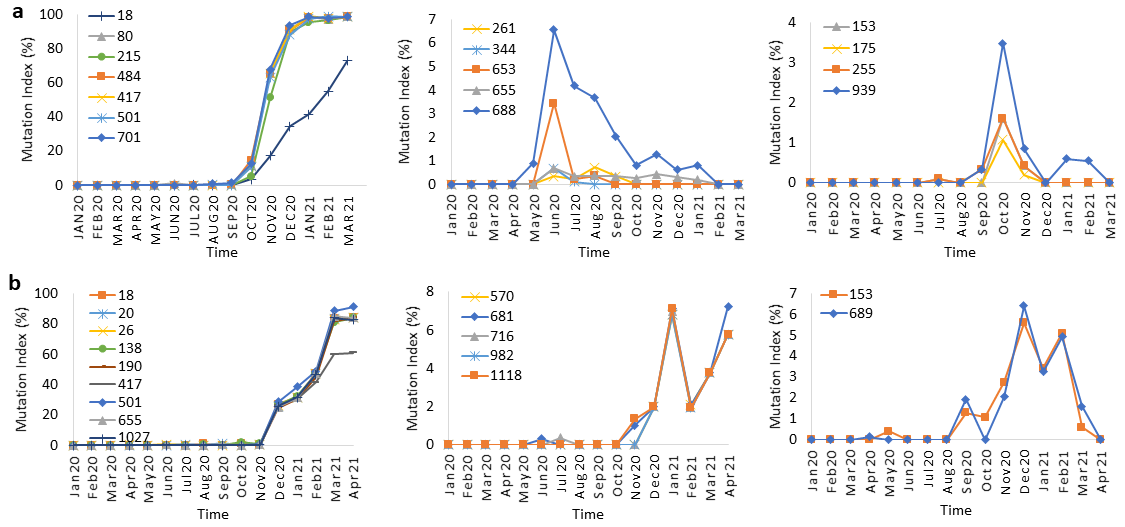


Fig S2. Mutation index data obtained from a) Italy and b) Spain.


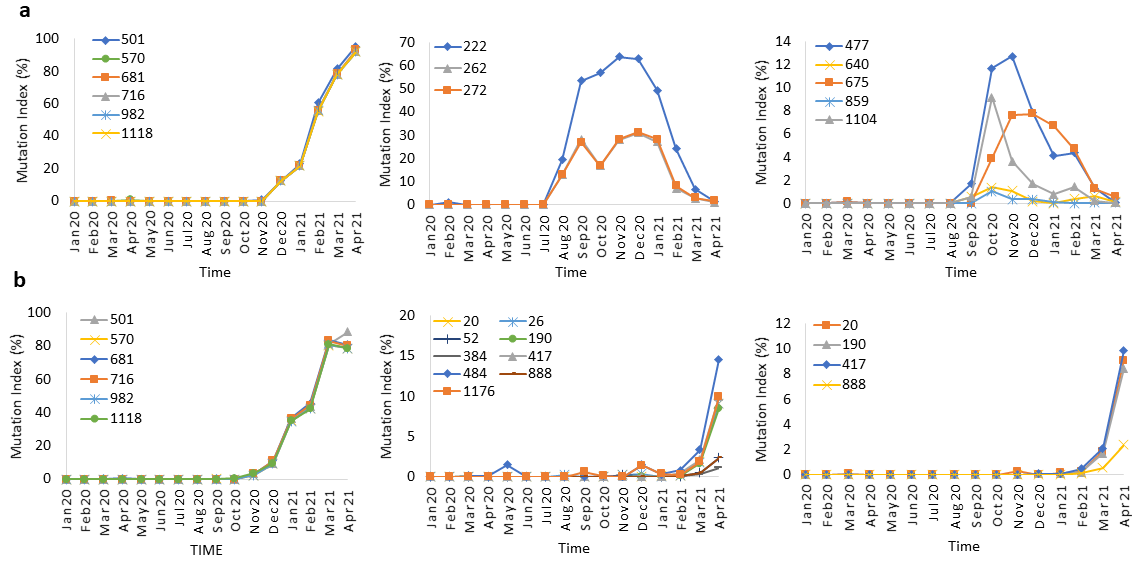


Sup. T1. Top 16 mutations in spike protein sequences from 12 different countries.


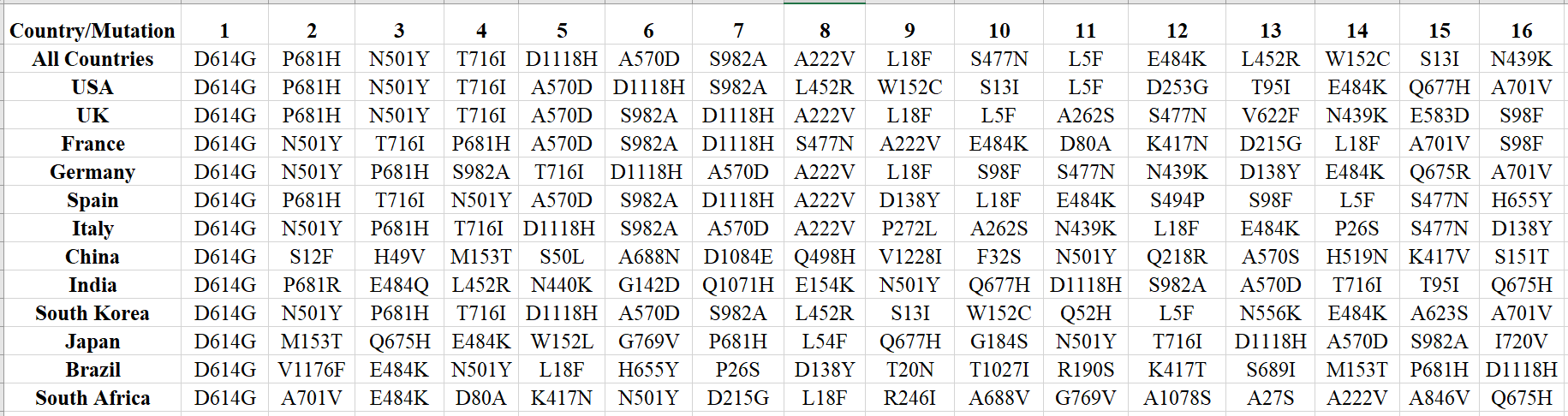


Sup. T2. Mutation indices for all 36 countries are shown in an Microsoft Excel sheet as supplementary material available at http://curie.utmb.edu/COVID19/.
